# Identification of deleterious variants associated with male infertility genes in a cohort of idiopathic hypospermatogenesis patients

**DOI:** 10.3389/frph.2024.1494585

**Published:** 2025-01-03

**Authors:** Nisha Sharma, Ashutosh Halder, Seema Kaushal, Manoj Kumar, Manish Jain

**Affiliations:** ^1^ Department of Reproductive Biology, All India Institute of Medical Sciences, Delhi, India; ^2^ Department of Pathology, All India Institute of Medical Sciences, Delhi, India; ^3^ Department of Urology, All India Institute of Medical Sciences, Delhi, India

**Keywords:** whole exome sequencing, male infertility, structural variants, azoospermia, hypospermatogenesis

## Abstract

**Introduction:**

Hypospermatogenesis is a common histopathological subtype of non-obstructive azoospermia and is characterized by a decrease in the total number of germ cells within the seminiferous tubule as a result of spermatogenic failure. Determination of genetic factors before intracytoplasmic sperm injection can prevent the inheritance of these factors, as hypospermatogenesis patients gives high successful sperm retrieval rate. This study aimed to identify the structural variants associated with idiopathic hypospermatogenesis (iHS) by analyzing patient cohorts diagnosed with azoospermia using whole exome sequencing.

**Methods:**

It is a hospital-based observational study in which patients reporting with azoospermia due to spermatogenic failure were recruited prospectively. Comprehensive clinical history, blood samples, semen analysis parameters, and reproductive endocrine evaluation reports of 51 hypospermatogenesis patients were collected. The known genetic causes were investigated using XY fluorescent *in situ* hybridization and Yq microdeletion for exclusion. Whole exome sequencing was performed, and the data of 42 iHS patients was analyzed to identify single nucleotide variants associated with diagnostically important male infertility genes.

**Results:**

Genomic analysis of SNVs identified rare deleterious candidate variants in *CFTR* (c.1265C>T; p.Ser422Phe), *CYP21A2* (c.955C>T; p.Gln319Glu), *SRD5A2* (c.737G>A; p.Arg245Gln), *LHCGR* (c.378A>C; p.Lys126Asn) and *AR* (c.2179C>A; p.Arg727Ser) genes associated with 7/42 idiopathic hypospermatogenesis patients. In silico analysis of variants shows deleterious and probably damaging effects on canonical transcripts of the genes.

**Discussion:**

This exploratory genomic analysis conducted on idiopathic hypospermatogenesis patients shows prevalence of rare deleterious candidate variants in genes associated with human male infertility. The candidate variants in idiopathic hypospermatogenesis patients are heterozygous and genotypically associated with syndromic male infertility. The symptomatic heterozygosity leading to mild spermatogenic failure resulting in hypospermatogenesis points towards a multifactorial etiology of the disease. This study justifies the importance of genetic screening of idiopathic hypospermatogenesis patients for the presence of structural variants in known human male infertility genes.

## 1 Introduction

Hypospermatogenesis (HS) is characterized by a morphological abnormality of the testicular germinal epithelium leading to reduced cellular density within the tissue (1). While all the developmental stages of the sperm germ cells are discernible, their overall number is notably reduced. The primary defect in HS lies within the stem cell population, making it a complex and multifactorial condition (2). Approximately 45% of patients exhibit identifiable causes, with the remainder being idiopathic (3). This abnormality is visible upon biopsy of the germinal epithelium of the testis. Additionally, sperm analysis reveals azoospermia or severe oligozoospermia in the patient with HS. Congenital causes of HS often stem from genetic abnormalities, including abnormalities in sex chromosome number or structure. Chromosomal rearrangements such as deletions, duplications, or translocations can disrupt gene dosage, particularly in regions critical for spermatogenesis on the Y chromosome. For instance, Klinefelter syndrome (47, XXY) is a common genetic cause of azoospermia (4). A syndrome exhibiting decreased male fertility as a clinical manifestation has been reported in a previous study done by Spela M. et al. (5). Mutations in genes that are crucial to spermatogenesis, such as *TEX11, HFM1, ATG4D, SYCP3*, and *DMRT1,* can disrupt germ cell development and function (6). Polymorphisms in genes such as follicle-stimulating hormone receptor *(FSHR*) and androgen receptor (*AR*) have also been implicated in HS (7). Nevertheless; from a treatment outcome point of view, HS gives the highest success rate upon sperm retrieval and hence is the testicular phenotype of highest value (8). The whole exome sequencing (WES) is a valuable tool for identifying genetic variants associated with idiopathic hypospermatogenesis (iHS), particularly single nucleotide variants (SNVs), due to its high sensitivity (9). This approach helps discover both known and novel mutations in HS patients, helping in the diagnosis and understanding of disease mechanisms.

This study aims to identify the genetic factors associated with iHS by using WES. The data generated from these analyses was used to identify the prevalence of structural variants in diagnostically important spermatogenesis-regulating genes, which will ultimately contribute to the understanding of male infertility (10). This will facilitate the development of more effective diagnostic strategies and personalized treatment modalities for HS patients.

## 2 Materials and methods

### 2.1 Patient recruitment and laboratory procedures

The study was conducted at the Department of Reproductive Biology, All India Institute of Medical Sciences, New Delhi, India, for three years from March 2020 to March 2023. Infertile male patients coming to the Department of Urology were recruited, and clinical history and physical examination were performed for each case. Informed written consent was obtained from the participants, and the study has been performed according to the Declaration of Helsinki. The research was conducted according to the guidelines of the institute ethical committee of the All India Institute of Medical Sciences in Delhi, India. The approval ID is IEC-151/06.03.2020, RP-17/2020. Hormonal evaluation of infertile men was performed using chemiluminescent microparticle immunoassay (CMIA). Quantification of serum follicle stimulating hormone (FSH), serum luteinizing hormone (LH), and serum testosterone was performed using 2nd generation Abbott laboratories' kits. Semen analysis was performed for each infertile man following WHO criteria 2021 (11). The ejaculate was collected in a preweighed container, and semen volume was calculated. A 30-min incubation for liquefication of the semen sample was done, and the pH was determined. The semen sample was heated in strong acid and resorcinol to measure fructose concentration. A 20 µl semen sample was prepared by carefully mixing the ejaculate and examined under 10× followed by 40× magnifications. For spermatozoa ≧101 per field at 40× magnification, a 1:20 dilution ratio was chosen to count sperm in 50 µl of well-mixed ejaculate and 950 µl of fixative. Microscopic counting was done in Neubauer's chamber. Cases with no sperm seen even after centrifugation and a sperm concentration <2.5 million/mL were considered azoospermia and oligozoospermia, respectively. The patients with obstructive causes were excluded, and a bilateral testicular fine needle aspiration evaluation was performed for a confirmatory diagnosis of NOA.

### 2.2 Identification of HS cases

Azoospermic and oligozoospermic men with normal or slightly altered reproductive endocrine parameters were advised for testicular fine needle aspiration (FNA) evaluation in order to be categorized as HS. The testicular FNA procedure, followed by reporting, was done at the Department of Pathology. In order to obtain the sample, a spermatic block containing 1% lignocaine was given to the HS patients. A 22-gauge needle attached to a 10 ml syringe on a plunger was used at two distinct locations to get the aspirates from the right and left testicles. A thread-like structure in the aspirate was used to prepare smears before staining. Every smear was confirmed for the presence of at least 2,000 cells. FNA from both testes shows a considerable decrease in the spermatogenic series of cells per Sertoli cell; however, maturation till spermatozoa is seen, suggesting HS.

### 2.3 Identification of iHS cases

A comprehensive clinical history of the HS patients was taken to investigate male factor infertility. Patients were excluded if they had conditions such as the presence of hydrocele or varicocele, history of testicular trauma, testicular maldescent, and history of radiotherapy, chemotherapy, or exposure to any reproductive toxin and orchitis. A blood sample of 51 HS patients (Supplementary Sheet 1) was collected in EDTA vials for DNA isolation using the QIAamp DNA Blood Mini Kit (QIAGEN, 51106) according to the manufacturer's instructions. The optical density of the isolated DNA was measured with a spectrophotometer (Nanodrop). Metaphase cell culture was conducted for 51 HS cases using a heparinized blood sample in karyotyping medium (Thermofisher Ref 12557-013). XY FISH analysis was performed on metaphase cells utilizing in-house X and Y centromere probes. Signals from X- and Y- centromeric probes were enumerated in a minimum of five metaphase cells. Multiplex sequence-tagged sites (STS) PCR analysis for Yq microdeletion was carried out for 46 HS cases using AZFa-sY84, sY86; AZFb-sY127, sY134; AZFc-sY254, sY255, and SRY primers. A PCR amplicon was run on a 4% agarose gel and visualized. The HS cases were considered idiopathic after excluding microdeletions of the Y chromosome found in 4 patients and aneuploidies of the sex chromosomes by FISH analysis, found on 5 patients. We finally selected a total of 42 iHS cases for the WES study.

### 2.4 WES and data analysis

Sequencing libraries were prepared using SureSelectXT Human All Exon (SSV5 + UTRs). The enriched DNA libraries were multiplexed by adding index tags by amplification, followed by purification. Prepared libraries were sequenced on an Illumina HiSeqX to generate 100X target coverage and 2 × 150 bp reads/sample. Approximately 75% of the sequenced bases had Q30 values. The sequence was processed to generate FASTQ files. Base trimming was performed using a custom script, and open-source software, such as cutadapt or fastq-mcf, was used for adapter trimming. Read alignment was performed using the HG19 version of the BWA aligner. PCR-duplicate reads were removed using the Picard toolkit. Reads were realigned around the known indels using the Genome Analysis Toolkit (GATK—Indel Realigner). Both haplotype caller and genotype caller variant files were merged into a single variant VCF file.

### 2.5 Variant filtering strategy

**Figure 1:**
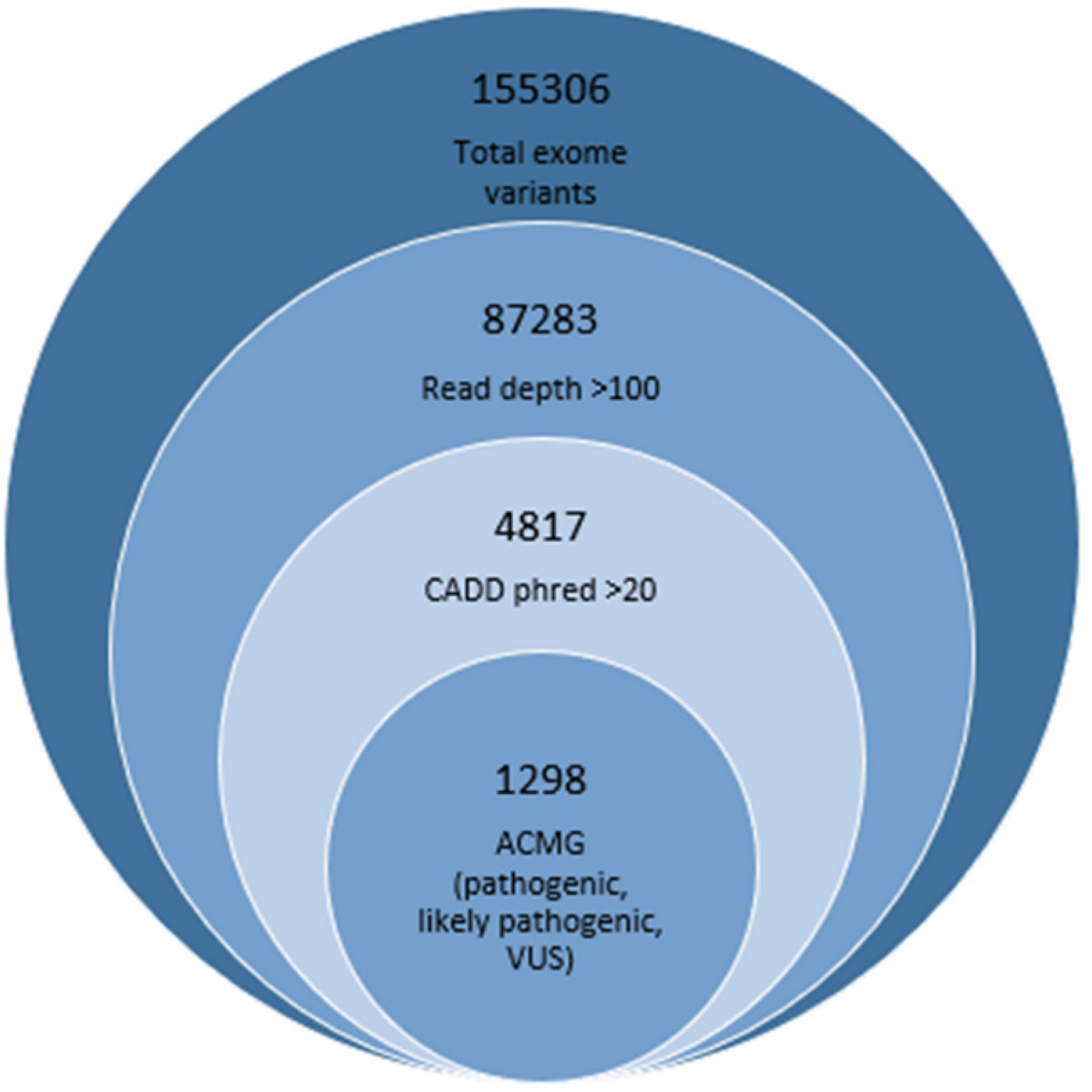
Variant filteration criteria showing number finally retained deleterious variants represented in smallest circle.

The variant filtering for SNV was performed by incorporating scores of various databases and software, including gnomAD, popmax, 1K genomes (for allele frequency), Sorting Intolerant From Tolrerant (SIFT), PolyPhen, mutation tester, Fathmm, variant Effect Scoring Tool (VEST), and combined annotation-dependent depletion (CADD) phred (for deleteriousness) (12). The read depth cutoff was considered >150 to avoid sanger validation. The variants reported as deleterious as per SIFT, allele frequency <0.01 as per 1K genome, and phred score >20 as per CADD were retained in the initial filtering. Only high and moderate impact (frameshift, stopgain, stoploss, startloss, missense) damaging variants of the exonic region with an ACMG classifier indicative of a pathogenic or likely pathogenic category were included in the final list as candidate variants. We also report the variants of unknown significance (VUS) as potential candidate variants. Figure 1 is showing the total number of variants found in one representative annotated file. The total number of variants was progressively reduced by applying the filtering criteria. Finally retained genes were manually checked for expression in testes from NCBI, and 120 high-probability human male infertility genes were included in the result section (10).

## 3 Results

### 3.1 Genetic exclusion of known causes

We performed XY-FISH analysis on both interphase and metaphase cells using X and Y centromere probes. Signals from X- and Y- centromeric probes were quantified in approximately 1,000 interphase cells and a minimum of 5 metaphase cells. Four cases of XXY males and one case of XX male were identified and subsequently excluded from further analysis. Furthermore, among the remaining cases, three patients with HS displayed microdeletions in the AZFc region while one patient exhibited a microdeletion in the AZFa region. The diagnostic importance of gonosomal abnormalities and Yq microdeletion in HS patients was determined and shown in Figure 1 in the later part of the results. The HS cohort consisted of 51 patients; however, after the exclusion of 9 patients due to gonosomal aberrations or AZF deletions, 42 iHS cases were subjugated to WES analysis. The database number (DBN), clinical findings and laboratory parameters are given in Supplementary Sheet 1.

### 3.2 Rare deleterious variants associated with male infertility genes

To identify the structural genomic variants in iHS patients, the whole exome data was filtered to identify deleterious variants with pathogenic, likely pathogenic, and VUS scores. It was observed that 16.6% (7 out of 42 iHS) of patients exhibited pathogenic or likely pathogenic SNVs in five diagnostically important human male infertility genes (Table 1). Among the observed candidate variants, one CFTR gene variant was identified as missense mutations with a likely pathogenic classification in two iHS patients. The variant c.1265C>T resulting in p.Ser422Phe protein change was associated with congenital bilateral aplasia of the vas deferens, as evidenced by their relationship with CFTR-related conditions. Similarly, one pathogenic variant in the CYP21A2 gene, characterized by a missense mutation (c.955C>T, p. Gln319Glu), was identified. The CYP21A2 heterozygous mutation was detected in two iHS patients and is linked to hyperandrogenism, non-classic type, due to 21-hydroxylase deficiency, thus suggesting its involvement in the manifestation of HS. The SRD5A2 heterozygous pathogenic variant was detected in one patient and is linked to pseudovaginal perineoscrotal hypospadias, which manifests infertility as a reproductive manifestation. Additionally, one pathogenic missense variant in the AR gene (c.2179C>A, p. Arg727Ser) was observed. We also report one VUS associated with the LHCGR gene, which has been reported to be related to Leydig cell hypoplasia with hypergonadotropic hypogonadism leading to infertility. These findings demonstrate the genetic heterogeneity underlying HS and highlight the importance of precisely investigating the human male infertility-linked genes in a cohort of male infertility due to HS. The iHS patients showing genotypic factors of male infertility were assessed for the clinical features and laboratory parameters in order to establish genotype-phenotype correlation (Table 2).

**Table 1:** Candidate variant associated with known human male infertility genes found in iHS phenotype.

Frequency in iHS cohort	Gene	Mutation type	HGVS	VAF	Variant classifiers (ACMG)	Previously reported condition	rs ID	Zygosity
*N* = 2	*CFTR* NM_000492.4	Missense	c.1265C>T p.Ser422Phe	0.000207	Likely pathogenic	Congenital bilateral aplasia of vas deferens from CFTR mutation (OMIM#277180)	rs201880593	Het
*N* = 2	*CYP21A2* NM_000500.9	Missense	c.955C>T p.Gln319Glu	–	Pathogenic	Hyperandrogenism, nonclassic type, due to 21-hydroxylase deficiency, (OMIM#201910)	rs7755898	Het
*N* = 1	*SRD5A2* (NM_000348.4)	Missense	c.737G>A p.Arg245Gln	0.000199	Pathogenic	Pseudovaginal perineoscrotal hypospadias, (OMIM#264600)	rs9332967	Het
*N* = 1	*LHCGR* (NM_000233.4)	Missense	c.378A>C p.Lys126Asn	0.000566	VUS	Leydig cell hypoplasia with hypergonadotropic hypogonadism, (OMIM#238320)	rs373456950	Het
*N* = 1	*AR* (NM_0000096473.9)	Missense	c.2179C>A p.Arg727Ser	0.0001415	Pathogenic	Androgen insensitivity (OMIM#312300)	rs781301298	Hemi

CFTR, cystic fibrosis transmembrane conductance regulator; CYP21A2, cytochrome P450 family 21 subfamily A member 2; SRD5A2, steroid 5 alpha-reductase 2; LHCGR, luteinizing hormone/choriogonadotropin receptor; AR, androgen receptor; HGVS c., human genome variation society coding DNA reference sequence; HGVS p., human genome variation society protein change; VAF, variant allele frequency; ACMG, American College of Medical Genetics and Genomics; OMIM, online mendelian inheritance in man; rs ID, reference single nucleotide polymorphism id; Het, heterozygous; Hemi, hemizygous.

**Table 2:** Clinical and laboratory investigation parameters of iHS patients associated with deleterious variants.

Parameter	Idiopathic hypospermatogenesis patient database number with gene variant detail						
	HS1 *CFTR* (rs201880593)	HS2 *CFTR* (rs201880593)	HS3 *CYP21A2* (rs7755898)	HS4 *CYP21A2* (rs7755898)	HS5 *SRD5A2* (rs9332967)	HS6 *LHCGR* (rs373456950)	HS7 *AR* (rs781301298)
Age (year)	34	30	27	41	37	28	24
Duration of infertility	Primary infertility for 6 years	Primary infertility for 4 years	Primary infertility for 3 years	Primary infertility for 18 years	Primary infertility for 8 years	Primary infertility fro 4 years	Primary infertility fro 2 years
BMI	25.5	27.4	24.2	29.9	26.7	25.3	23.4
Semen analysis	Severe Oligo., Fructose + ve, Alkaline	Azoospermia, Fructose + ve, Alkaline	Azoospermia, Fructose + ve, Alkaline	Severe Oligo., Fructose + ve, Alkaline	Azoospermia, Fructose + ve, Alkaline	Azoospermia, Fructose + ve, Alkaline	Azoospermia, Fructose + ve, Alkaline
Serum T	5	3.64	2.65	4.82	4.77	3.26	4.2
Serum FSH	7.59	19.69	28.95	24.67	3.7	6.8	10.48
Serum LH	4.37	5.35	6.63	11.88	6.08	3.45	2.67
Testicular Vol. (ml)	R = 15 L = 15	R = 12 L = 12	R = 12 L = 12	R = 10 L = 10	R = 15 L = 15	R = 15 L = 12	R = 12 L = 10
XY-FISH	*N*	*N*	*N*	*N*	*N*	*N*	*N*
Yq MD	*N*	*N*	*N*	*N*	*N*	*N*	*N*

BMI, body mass index; Serum T, serum testosterone (reference range = 1.42–9.23 ng/ml); Serum FSH, serum follicle stimulating hormone (reference range = 0.95–11.95 mIU/ml); Serum LH, serum leutenising hormone (reference range = 0.57–12.07 mIU/ml); Testicule vol., testicular volume right and left (Azoospermia, no sperm seen even after centrifugation; severe oligo, severe oligozoospermia (reference range <2.5 million sperm in the ejaculate seen after centrifugation); N*,* normal phenotype.

### 3.3 In-silico prediction

Effect prediction to identify the deleteriousness of variants on protein structure and function was performed using in-silico tools (Table 3). SIFT, PolyPhen, and Fathmm are used to predict the deleteriousness or probably damaging effect of variants. SIFT score 0 to 0.05 indicates deleteriousness, PolyPhen score near 1 is considered probably damaging, and Fathmm score above 0.5 indicates deleteriousness. The disease-causing potential of the variants was assessed with a mutation tester (score ≧0.5). The pathogenicity was assessed with VEST (close to 3) and CADD phred (score >20). We considered the effect of variants to be damaging for protein structure and function if one or more tools outcome score is potentially detrimental in nature. The deleterious candidate variants were found in canonical transcripts of the genes, hence affecting the protein function directly.

**Table 3:** Assessment of effect of variants on protein using in-silico prediction tools.

Variant name	In-silico prediction tool						
	SIFT	PolyPhen	Mutation tester	Fathmm	VEST	CADD phred	Canonical
*CFTR* (NM_000492.4: c.1265C>T)	Tolerated	Benign	Neutral	Deleterious	–	Deleterious	Yes
*CYP21A2* (NM_000500.9: c.955C>T)	–	–	A	Deleterious	–	Deleterious	Yes
*SRD5A2* (NM_000348.4: c.737G>A)	Deleterious	Probably damaging	–	–	Neutral	Deleterious	Yes
*LHCGR* (NM_000233.4: c.378A>C)	Deleterious	Benign	Deleterious	Deleterious	Neutral	Deleterious	Yes
*AR* (NM_0000096473.9: c.2179C>A)	Deleterious	Probably damaging	Deleterious	Deleterious	Neutral	Deleterious	Yes

SIFT, sorting intolerant from tolerant; PolyPhen, polymorphism phenotyping; Fathmm, functional analysis through hidden Markov models; VEST, variant effect scoring tool; CADD, combined annotation-dependent depletion.

### 3.4 Determination of diagnostic yield

**Figure 2:**
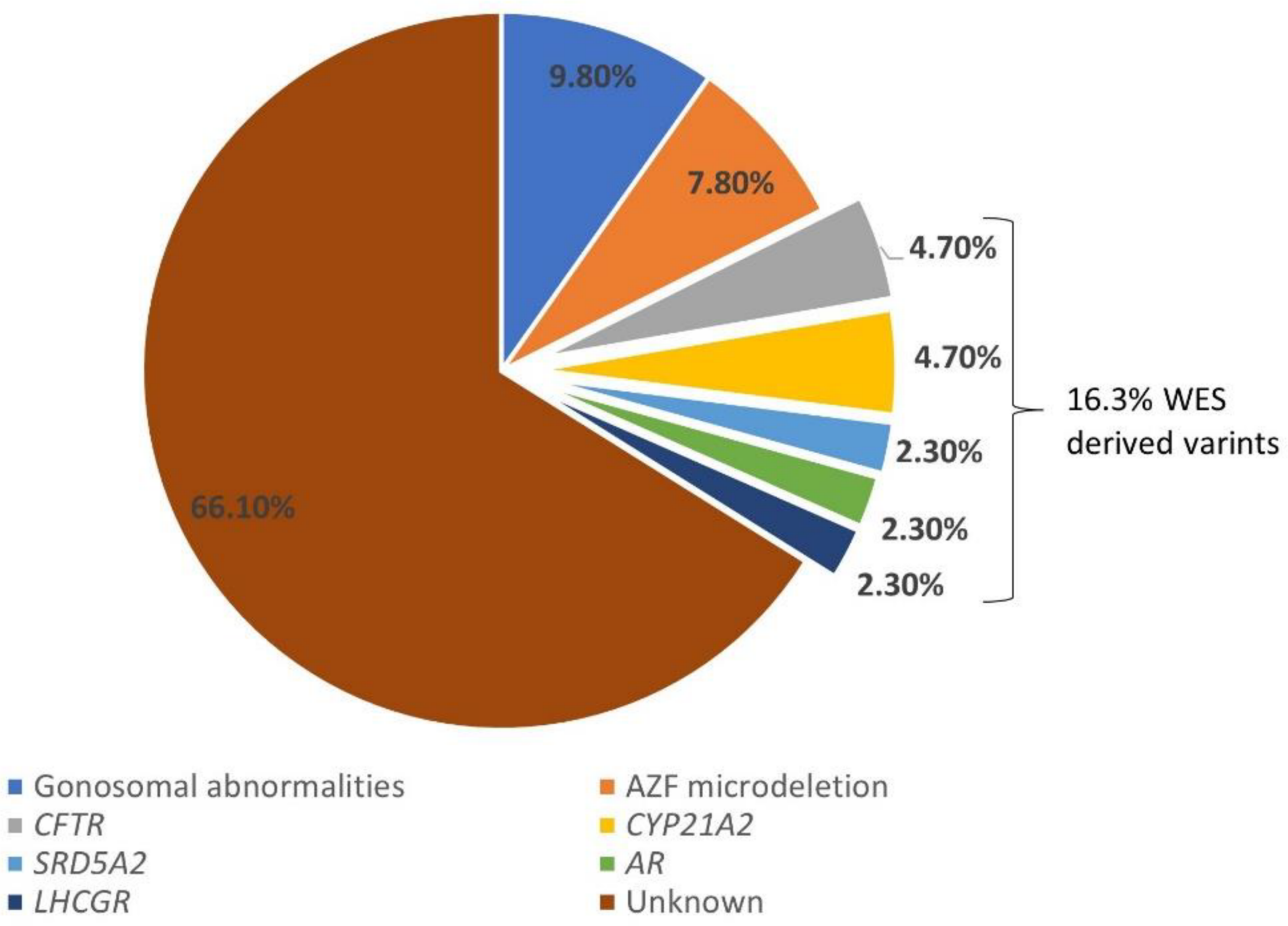
Dignostic yield of genetic test and whole exome sequencing in HS patients.

Diagnostic rate refers to the percentage of diagnostic tests or procedures that successfully identify a specific condition, disease, or disorder. The diagnostic yield of genetic testing with different etiologies was assessed in iHS patients. Gonosomal abnormalities accounted for 9.8% (5 out of 51 recruited HS cases) of cases, while AZF microdeletions were identified in 7.8% (4 out of 51 recruited HS cases) of patients. CFTR and CYP21A2 mutations were detected in 4.7% (2 out of 42 recruited HS cases) of cases. The SRD5A2, LHCGR, and AR mutations were found in 2.4% (1 out of 42 recruited HS cases) of patients (Figure 2). Notably, a significant portion, constituting 66% (35 out of 51 recruited HS cases), remained of unknown etiology despite genetic testing and WES analysis. These findings show the complexity and heterogeneity of genetic factors contributing to HS.

## 4 Discussion

Azoospermia due to primary testicular failure is a difficult condition to manage at an andrology clinic and hence gives poor treatment outcomes. The histopathological subtype of azoospermia producing the best treatment outcome is HS, the reason being the presence of a noticeable number of germ cells inside the seminiferous tubule. The sperm retrieval in HS patients gives up to a 90% success rate, which is highest among all the subtypes of NOA (13). The congenital causes leading to HS are mainly genetic; therefore, tests to identify the known and unknown causative factors could provide insight into the diagnostic yield of existing genetic tests together with WES for HS patients. The known genetic factors include abnormalities of sex chromosome number (aneuploidy), which is identified using FISH or karyotype, and structural deletions are identified using Yq microdeletion (14). With the advent of WES technology, the smallest structural variants, like SNVs, are discernible in idiopathic male infertility patients. Oud and colleagues mentioned genes (AR, APOA1, TEX11, TEX15, AURKC, CFAP43, CFAP44, DNAH1, DPY19L2, SUN5, WDR66, CCDC39, CCDC40, CFAP69, LRRC6, PIH1D3, PKD1, PMFBP1, NR5A1) that have reached the moderate or high grade of clinical evidence for the diagnosis of NOA (15). Currently, substantial literature suggesting the prevalence of genomic causes in specifically HS patients is not available. Categorization of monogenic causes in HS, sertoli cell only syndrome, and maturation arrest has not been explicitly mentioned in the literature; therefore, strong evidence for genomic causes of HS remains to be explored further.

In this study, we found 9.8% of HS patients with gonosomal abnormalities in XY-FISH analysis. Klinefelter syndrome (KS) is a chromosomal aneuploidy (47XXY) that causes impaired spermatogenesis to a variable extent. It is the most commonly known genetic cause of azoospermia, accounting for up to 14% of all cases. The 47, XYY Syndrome is another anomaly with testicular histology that mainly shows maturation arrest (MA) or Sertoli cell only syndrome (SCOS) subtypes (16). Small foci of decreased number of germ cells in the testes can be present in the patients with chromosomal abnormalities indicative of KS (17). Moreover, in our study, all four patients with KS were found to have high serum FSH levels indicative of primary testicular failure (Supplementary Table 1). Another genetic test performed to discern known genetic causes in azoospermia is Yq microdeletion. The Y chromosome contains genes critical for spermatogenesis (AZFa, AZFb, and AZFc regions of the q arm). AZFc is the largest region, measuring at 4.2 Mb, followed by AZFb at 3.2 Mb, and finally AZFa at 792 Kb (18). We found 7.8% of HS patients to be associated with AZF microdeletion, which is similar to the previous study done on the Indian population of azoospermia patients (19).

In the current study we performed WES and explored the genes previously linked to human male infertility to find out the frequency of deleterious variants associated with iHS patients. We found heterozygous variants in CFTR, CYP21A2, SRD5A2, and LHCGR genes that could cause a mild spermatogenic failure seen in HS. The genetic heterogeneity observed in the variations demonstrates the importance of investigating spermatogenesis-linked genes in a cohort of male infertility due to a likely phenotype of hypospermatogenesis, a relevant condition in the transmission of genetic mutations to offspring obtained through assisted reproduction treatments. A recent study of Quarantani et al. on 99 idiopathic azo/oligozoopsermic men showed that when a variant prioritization to whole exome sequencing (WES) is applied, a considerable number of Mendelian causes of infertility can be uncovered even in a small cohort of patients (20). The symptomatic heterozygosity found in male infertility patients in our study manifests as a mild form of spermatogenic failure. The clinical presentation of iHS patients associated with rare deleterious candidate SNVs shows heterozygous phenotypic effect causing impaired spermatogenesis, which is reflected by higher FSH levels in 3 iHS (HS2, HS3, and HS4) patients. Symptomatic heterozygotes have been observed in various disease categories, such as neurological, neuromuscular, hematological, and pulmonary conditions, with moderate and non-specific symptoms manifesting at a later stage in life. Possible causes encompass undiscovered deep-splicing variations, genetic and environmental modifiers, digenic/oligogenic inheritance, skewed methylation patterns, and mutational burden (21, 22). The multifactorial etiology of idiopathic male infertility associated with mild spermatogenic failure should be simultaneously explored for epigenomic and environmental causes to completely justify the genotype-phenotype correlation.

Houston et al. published a list of validated monogenic causes of human male infertility with further categorization into isolated infertility, syndromic infertility, and reproductive system/endocrine disorders-related gene variants (10). We found variants in genes categorized into isolated infertility (AR and CFTR), reproductive disorders (SRD5A2), and endocrine disorders (LHCGR and CYP21A2). Notably, the diagnostic rate observed in the current study exceeds the reported diagnostic rates for genetic tests in isolated male infertility, which typically range between 4% and 9.2% according to previous studies (23, 24). The CFTR heterozygous mutations detected in our study could be associated with a partial obstruction or other related conditions not screened in two iHS patients (HS1 and HS2). However, a study done by Sharma H. et al. on Indian men with spermatogenic failure without congenital bilateral absence of vas deferens found an increased frequency of probably damaging CFTR gene mutations (25). In a recent study, Qiang Li et al. found the CFTR gene mutation to be the associated etiological factor in patients with oligoasthenospermia other than congenital absence of vas deferens in Chinese patients of male infertility (26).

The aforementioned results provide an adequate basis to investigate the presence of deleterious heterozygous mutations in the CFTR gene in iHS patients prior to undergoing sperm retrieval. It is important to understand the molecular relationship between CFTR gene variations and spermatogenesis in order to better comprehend their contribution to HS. The most frequently seen correlation of CFTR gene mutations is with the congenital absence of vas deferens, which results in obstructive azoospermia.

In a Chilean study on males with congenital adrenal hyperplasia, a severe homozygous mutation resulting in p.Gln318* was found with no evidence of infertility, whereas we found two heterozygous variants resulting in p.Gln319Glu as protein functional loss leading to azoospermia (27). The detailed pathogenic mechanism of variants associated with CYP21A2 needs to be explored in males, whereas in females it is reported to cause infertility (28). Association studies on polymorphism of SRD5A2 and male infertility and seminal parameters have been studied in various populations, and conflicting results have come out (10, 29). We found a pathogenic variant that is rare in the population database and might be responsible for spermatogenic failure in iHS patients. The variant of uncertain significance associated with the LHCGR gene was found deleterious as per SIFT and CADD and therefore displays evidence of a detrimental effect on LH receptor protein function and leading to spermatogenic failure in HS. We also found one hemizygous mutation in an X-linked AR gene as definitive evidence causing spermatogenic failure in HS. In an iHS patient with an AR gene mutation, the testosterone level was found to be 15.60 nmol/L, and the androgen sensitivity index calculated as LH (mIU/ml) × T (nmol/L) was found to be 41.56 IU × nmol/L2. This finding is contradictory to a recent study of Rocca et al., who studied 8,224 idiopathic azo/oligozoospermic patients and observed that a testosterone level of ≥15.38 nmol/L and an androgen sensitivity index (ASI) between 230.9 and 405.6 IU × nmol/L2 can discriminate patients in risk of harboring mutations in the AR gene (30). The genotype-phenotype relationship of gene variations, including CYP21A2, LHCGR, and AR, has been found to exhibit degree of variability. The diseases that are related to these gene mutations are complex, and the aggravation of symptoms that lead to HS may be the result of the influence of environmental factors.

The diagnostic rate from analysis of genetic and genomic factors gives 16% (7 out of 42 recruited HS cases) yield in identification of etiological factor for spermatogenic failure in HS patients. The process of testis development is governed by approximately 2,300 genes, with at least 900 of these genes being responsible for regulating spermatogenesis (31). Evaluating a limited number of genes could give a high diagnostic yield only if that variant is repeatedly found to be associated with spermatogenic failure. This poses a need for more studies for characterization of causative variants in male infertility. Furthermore, the diagnostic yield based on currently known causative genes associated with male infertility may not be sufficiently robust. Further exploration is needed to identify causative variants comprehensively.

## 5 Conclusions

A phenotype-based strategy to elucidate and classify the multifactorial etiology of male factor infertility has the potential to enhance treatment outcomes. In this study, an exploratory investigation of HS patients was conducted utilizing the WES technique. The objective was to identify potential candidate gene variants associated with this condition. Analysis of genomic factors in HS patients illustrated rare deleterious variants in key genes such as CFTR, CYP21A2, LHCGR, SRD5A2, and AR. The symptomatic heterozygosity leading to mild forms of spermatogenic disruption in HS patients is identified in the study. The study demonstrated a higher diagnostic yield of genetic testing in HS patients compared to reported rates in isolated male infertility.

## 6 Limitation

The study would have been improved by including a control group, although obtaining a sufficiently powered and well-matched cohort of control samples was expensive and time-consuming. Due to the limited occurrence of the iHS subtype of NOA, we were only able to conduct a whole WES study on a small cohort of 42 patients. All 42 iHS patients were gathered in a planned manner starting from the day when ethical approval was obtained, as there is no biobanking facility at the Institute to obtain stored DNA samples. Blood samples used for WES analysis might not be a representative of all variations in spermatogonia. Performing WES on sperms retrieved from iHS samples, if possible, can result in identifying more number of causative variants related to spermatogenesis.

## Data Availability

The datasets presented in this study can be found in online repositories. The names of the repository/repositories and accession number(s) can be found in the article/Supplementary Material.
